# A Typology of Livable Communities and Older Adults’ Health in the U.S

**DOI:** 10.3390/ijerph22050676

**Published:** 2025-04-25

**Authors:** Kyeongmo Kim, Denise Burnette, Sol Baik, Seon Kim

**Affiliations:** 1 School of Social Work, Virginia Commonwealth University, Richmond, VA 23284, USA; jdburnette@vcu.edu; 2 Weldon Cooper Center for Public Service, University of Virginia, Charlottesville, VA 22903, USA; sbaik@virginia.edu; 3 Department of Social Welfare, Sunchon National University, Suncheon, Jeollanamdo 57922, Republic of Korea; seonkim@scnu.ac.kr

**Keywords:** age-friendly community, livable community, neighborhood typology, person–environment fit

## Abstract

Neighborhoods with high-quality built environments and social environments are associated with older adults’ well-being. However, research on the complex interplay of neighborhood types and health outcomes is limited, as is the role of functional limitations. This study aims to: (1) identify neighborhood types, (2) explore the association of neighborhood type and older adults’ health, and (3) assess whether functional status affects this association. We merged data from the 2017 AARP Age-Friendly Communities Surveys and the Livability Index. Our sample included 3211 adults aged 65 and older; the majority (59%) were female. Participants identified as non-Hispanic White (81%), Hispanic (8%), Black (6%), and a member of another racial/ethnic group (2%). Employing latent class analysis, we identified a four-class model of neighborhood types: “Connected yet Limited Services”, “Service Integrated”, “Healthy Environment Zones”, and “Supportive Social Engagement”. Older adults in “connected yet limited services” and “service-integrated” neighborhoods had worse self-rated health than those in “supportive social engagement” neighborhoods, especially among those who reported functional limitations. Our findings indicate that older adults with functional limitations particularly benefit from neighborhoods with robust health support and social engagement opportunities, highlighting the importance of designing inclusive and adaptable age-friendly environments to address diverse and changing needs.

## 1. Introduction

The quality of neighborhood environments is essential to older adults’ well-being, not least because they are more likely than other age groups to spend time in their neighborhoods [1]. Living in a neighborhood with a well-developed built environment and a positive social environment is strongly associated with better quality of life [2,3]. Conversely, physical and social risks associated with poor neighborhood environments lead to worse physical and mental health outcomes [4]. However, the findings of the existing research on the association of neighborhood characteristics and outcome variables are inconsistent.

For instance, Choi found that housing and outdoor spaces were significant factors for aging in place [5], yet Kim et al. reported that these same environmental features had low relevance for older adults’ health [6]. Meeks argues that these discrepancies may stem from the use of different outcome variables, which highlights the need for multiple outcome measures [7]. It is also plausible that these studies failed to account for neighborhood complexity. Neighborhood quality is typically more multifaceted than either extreme scenario. For example, a neighborhood may offer high-quality transportation but lack affordable housing options.

Research on this type of interplay between type of neighborhood and older adults’ health outcomes is limited, and few studies have examined the potential role of functional limitations in this association. The threefold purpose of the current study is to: (1) identify neighborhood types based on the World Health Organization’s age-friendly communities (AFC) framework [8], (2) explore the association of neighborhood type with older adults’ self-rated health, and (3) assess whether the association between neighborhood types and self-rated health varies by functional limitation.

### 1.1. Neighborhood Types and Health

The WHO developed the Age-Friendly Cities and Communities (AFC) framework in 2007 [8,9]. AFCs are designed to provide older adults with financially, physically, and culturally accessible resources and activities. They achieve these goals through policies, systems, and services that aim to remove barriers to access and engagement. The framework represents a significant milestone in promoting environments that support active and healthy aging.

Academic and policy scholars have since reached more of a consensus on basic features of the framework, which conceptualizes AFCs in terms of three key determinants of active aging: the built environment, social environment, and service environment. The built environment encompasses critical physical infrastructure (e.g., outdoor spaces, transportation, and housing) for mobility, safety, and social participation. The social environment considers community interactions and relationships that are essential to physical and psychological well-being, including social participation, respect, social inclusion, civic participation, and employment.

Finally, the service environment focuses on accessibility and the inclusivity of, for example, communication and information services and community support and health services that are important to older adults’ quality of life. Conceptualizing the interactions between these three domains of the neighborhood environment permits us to identify unique attributes that characterize types of neighborhoods, adding granularity to the current understanding of the complex mechanisms through which AFCs promote active aging and inclusive societies [10].

Scholarly work on the impact of AFCs increasingly focuses on whether neighborhood types are associated with older adults’ health and mental health outcomes [11], and a growing body of evidence supports such a relationship [12]. Features of built environments, such as green spaces, walkways, safety, and street lighting, are associated with more physical activity [11,13,14], better self-rated health [15,16,17], and psychological well-being [18]. Similarly, Lowen et al. found that older adults identified access to services, such as shopping, healthcare, and social clubs or community centers, as key components of good quality of life [19].

A few studies that examine the dimensions of AFCs provide valuable insights into their associations with well-being outcomes for older adults [16,20,21]. Using the Age-Friendly City Scale to assess different AFC domains, Au et al. documented the essential role of transportation and social and civic participation in older adults’ life satisfaction [20]. Similarly, Park and Lee found that supportive physical and social environments in AFCs can improve life satisfaction, particularly for vulnerable subgroups of older adults, such as those who live alone or have lower incomes [21]. Older adults who resided in highly age-friendly communities were also more likely to anticipate staying in their community as they age [22]. These studies underscore the importance of understanding and articulating neighborhood types within AFCs and of systematically assessing their impact on older adults’ health outcomes for policymakers and other stakeholders in age-friendly initiatives.

### 1.2. Person–Environment Fit

The Person–Environment Fit Model is an apt framework for understanding the health impacts of neighborhood environments for older adults. This model, developed by Lawton and Nahemow, describes how well-being and health outcomes depend on the “fit” between an individual’s competencies (i.e., physical and cognitive abilities) and their surrounding environment. As older adults are more likely to face declines in mobility, cognitive function, or physical strength, the supportiveness of their neighborhood environments is a key factor in maintaining health and independence [23]. Neighborhoods with accessible health services, safe transportation, and opportunities for social engagement can provide a “good fit”, meaning the environment is well-aligned with their capacity to maintain independence and social connections. Conversely, neighborhoods that lack these features may contribute to social isolation, physical inactivity, and worsened health outcomes.

The current study aligns with Lawton’s model of person–environment fit in developing a typology of neighborhoods and examining how different types of neighborhoods are associated with older adults’ health outcomes. Furthermore, individuals seek to optimize the balance of personal competence and environmental demands. We thus hypothesize that, when personal competence is low, environmental pressures will have a stronger impact on self-rated health and, conversely, environmental pressure will be less consequential for individuals with high personal competence. These questions underscore the importance of developing and sustaining livable communities that enhance older adults’ health and well-being by fostering a positive person–environment fit.

Using data from the 2017 AARP Age-Friendly Communities (AFC) Surveys and the AARP Livability Index, this study draws on the WHO AFC framework to: (1) identify distinct neighborhood types; (2) determine the demographic characteristics associated with different neighborhood types; (3) examine the association of neighborhood types and self-rated health, controlling for relevant individual characteristics; and (4) assess whether the relationship of neighborhood type and self-rated health differ according to functional limitation status.

## 2. Materials and Methods

### 2.1. Data Sources

We merged data from the 2017 AARP Age-Friendly Communities (AFC) Surveys and the 2017 AARP Livability Index. The AFC Surveys aim to identify the needs of older adults in order to facilitate their ability to age in place [24]. The AARP research teams collected data in 13 U.S. metropolitan areas based on demographic composition and geographic location. The survey involved 30-minute telephone interviews with adults aged 45 and older from June 2017 through September 2017 (N = 6670). Following the American Association for Public Opinion Research, the team reported three response rates: average participation (49.1%), refusals (11.3%), and non-responses (4.4%). The AARP provides comprehensive details on the survey methodology by area [25].

The AARP created the Livability Index as a tool to evaluate how well neighborhoods support residents’ quality of life, with a focus on older adults [26]. By assessing neighborhoods and communities in terms of the essential services and amenities that have the greatest impact on residents’ lives, the Livability Index permits an overview of how well a community supports its residents [26]. With 61 indicators, the index measures community support across seven key areas: housing (affordability and access), neighborhood (access to life, work, and recreation), transportation (safe and convenient options), environment (clean air and water), health (preventive measures, access to healthcare, and quality of care), engagement (civic and social involvement), and opportunity (inclusion and possibilities). Indices range from 0 to 100, with higher scores indicating more favorable neighborhood conditions. The index is widely used in research and policy to understand how neighborhood conditions affect health [27], to assess urban quality of life [28,29], and to inform urban planning [30].

### 2.2. Sample

Our sample comprised respondents who met the following criteria: (a) aged 65 and older residing in the United States and (b) living in an area with a valid livability score and corresponding zip codes. We excluded individuals who were under 65 years of age (n = 2929), living in Puerto Rico (n = 504), and from areas that lacked a livability score (n = 26). Our analytic sample consists of 3211 adults aged 65 years and older (65–74 years (58%), 75–85 years (28%), and over 85 years (15%)) in 253 zip codes. Fifty-nine percent were female. Most were non-Hispanic White (81%), followed by Hispanic (8%), Black (6%), and other racial/ethnic groups (2%: Asian, Native American/Alaskan/Hawaiian).

### 2.3. Measures

#### 2.3.1. Livable Community

We used the AARP Livability Index, which evaluates the age-friendliness of neighborhoods. In this study, we define a neighborhood as a geographic area identified by zip codes. We operationalize neighborhood livability using seven different index scores within the corresponding zip code: housing, neighborhood, transportation, environment, health, engagement, and opportunity. Indices range from 0 to 100, with higher scores indicating more favorable neighborhood conditions. Communities that score above 50 are deemed to be above average, while those scoring at or below 50 are either average or below average [25]. This approach captures unique attributes and localized factors associated with older adults’ health.

#### 2.3.2. Self-Rated Health

We assessed self-rated health as a response to the question, “In general, would you say your health is …” Response options ranged from 1 (poor) to 5 (excellent), with higher scores indicating better self-rated health (*M* = 3.47, *SD* = 1.10, range: 1–5). We treated this measure as continuous, with a skewness of −0.37.

#### 2.3.3. Covariates

We adjusted for sociodemographic and health-related factors associated with self-rated health. Sociodemographic variables included age (1 = 65–74, 2 = 75–84, 3 = 85 and older), sex (0 = male, 1 = female), race/ethnicity (1 = White, non-Hispanic; 2 = Black, non-Hispanic; 3 = Hispanic; and 4 = other race/ethnicity), level of education (0 = less than college degree, 1 = Bachelor’s degree or above), income, employment status (0 = no, 1 = yes), homeownership (0 = no, 1 = yes), length of residence in current community (at least five years: 0 = no, 1 = yes), and living arrangement (0 = living alone, 1 = living with spouse or partner). Health-related variables included functional limitations (0 = no, 1 = yes) and the current family caregiving status (0 = no, 1 = yes). We also incorporated a measure of social interaction, assessed as the frequency of contact with family, friends, or neighbors who do not reside with the respondent, using a scale from 0 (never) to 6 (every day).

### 2.4. Data Analysis

We used latent class analysis (LCA) with a distal outcome to investigate the association between neighborhood type and self-rated health, adjusting for individual sociodemographic and health-related characteristics. First, we used LCA to identify types of livable communities. LCA is useful for revealing unobserved heterogeneity by discerning typologies from a set of indices [31]. To determine the structure and number of neighborhood types, we estimated a series of unconditional latent class models using seven livability indices. We assessed fit indices for each model to determine the optimal class solution, which included the Akaike Information Criterion (AIC), the Bayesian Information Criterion (BIC), and the Vuong–Lo–Mendell–Rubin adjusted likelihood ratio test (VLMR-LRT). We also considered the substantive interpretability of the identified classes and the number of neighborhoods across each class. Next, we evaluated the distinctiveness of the identified classes using average posterior probabilities (AvePP) greater than 0.70 [32] and conditional item probabilities exceeding 0.70 and below 0.30 [31].

We used bivariate analyses to examine sample statistics according to the neighborhood types we identified through LCA. Finally, we examined the association between neighborhood types and self-rated health, while adjusting for individual characteristics, using the Bolck, Croon, and Hagenaars (BCH) method [33,34,35]. This approach preserves consistent class membership while addressing classification uncertainty, thereby ensuring stable class formation [31]. Apart from income (13%), missing data ranged from 0 to 3%. We confirmed that the missing data were missing at random, indicated by a non-significant result from Little’s MCAR test. We employed the full information maximum likelihood estimation method, which is highly effective for addressing missing data. All data were analyzed using Mplus version 8.4 [36].

## 3. Results

### 3.1. Livable Neighborhood Types

Table 1 presents the model selection criteria for classifying neighborhood types. Fit indices indicated a preference for a five-class model; however, the reductions in both AIC and BIC were relatively modest compared to earlier models. Considering previous literature and the need for interpretability, we retained a four-class model, which offered greater conceptual clarity. Average posterior probability values exceeded the >0.70 threshold (ranging from 0.96 to 0.98).

**Figure 1:**
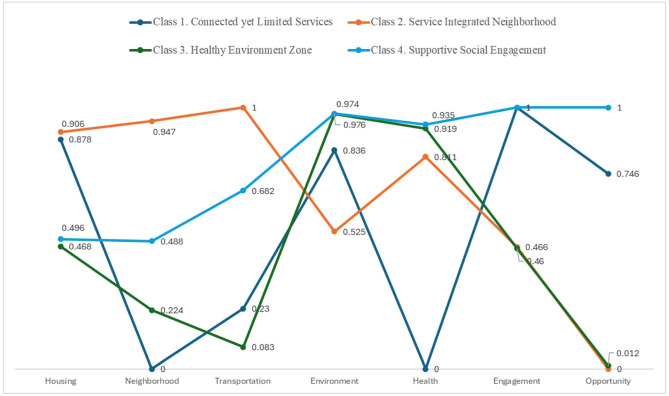
Patterns of neighborhood types.

Figure 1 presents the identified neighborhood types. Class 1, which comprised 15% of the sample, was designated as “Connected yet Limited Services”. This class is characterized by neighborhoods with strong housing, natural environments, social engagement, and opportunities, but limited neighborhood infrastructure, transportation, and health services. Class 2, labeled as “Service-Integrated Neighborhood” (28%), includes areas with a well-developed built environment—encompassing housing, neighborhood, and transportation—and health services, though the natural environment and social engagement are comparatively weak. Class 3, termed the “Healthy Environment Zone” (24%), describes neighborhoods with a high-quality natural environment and robust health services but limited built and social environments. Finally, we labeled Class 4, the largest group (33%), as “Supportive Social Engagement”. This class is characterized by neighborhoods with a quality natural environment, health services, and strong social engagement, but a limited built environment.

### 3.2. Comparison of Background Characteristics According to Livable Neighborhood Types

Table 2 presents sample characteristics for each neighborhood type. Adults aged 85 and older were more likely than younger age groups to reside in neighborhoods characterized by supportive social engagement (*x*^2^ = 18.79, *p* = 0.005). Older White adults predominated in neighborhoods with either “connected yet limited services” or “supportive social engagement”, and older Black adults were more likely to live in “connected yet limited services” or “service-integrated” neighborhoods. In contrast, older Hispanic adults were more often found in “service-integrated” neighborhoods and the “healthy environment zone” (*x*^2^ = 216.32, *p* < 0.001). Older adults with higher socioeconomic status were more likely to reside in neighborhoods offering more services and support. College graduates (*x*^2^ = 58.81, *p* < 0.001) were more likely to reside in “supportive social engagement” neighborhoods than in “connected yet limited services” neighborhoods, a pattern also observed for income level (*F* = 21.77, *p* < 0.001). No significant differences in neighborhood type were found in terms of sex, living arrangement, employment, social interaction, or caregiving status.

### 3.3. Livable Community Type and Health and the Role of Functional Limitations

After identifying the unconditional class, we conducted a latent class analysis (LCA) with a distal outcome using the BCH method. Table 3 presents findings on the relationship between neighborhood types and self-rated health, adjusted for individual characteristics. Compared to older adults residing in “supportive social engagement” neighborhoods, those in neighborhoods with “connected yet limited services” (b = −0.21, *p* < 0.001) and those in “service-integrated” neighborhoods (b = −0.16, *p* < 0.001) reported worse self-rated health. We found no statistically significant difference between the “healthy environment zone” and the “supportive social engagement” neighborhood types.

Compared to individuals aged 85 and older, younger adults reported worse self-rated health, as did Black (b = −0.22, *p* = 0.001) and Hispanic (b = −0.15, *p* = 0.040) older adults compared to White older adults. Older adults with a college degree (b = 0.14, *p* < 0.001), higher income (b = 0.07, *p* < 0.001), employment (b = 0.20, *p* < 0.001), homeownership (b = 0.12, *p* = 0.019), and greater social interaction (b = 0.06, *p* < 0.001) reported better self-rated health. Conversely, those individuals who were living with a spouse or partner (b = −0.12, *p* = 0.002) and those with functional limitations (b = −0.95, *p* < 0.001) reported lower self-rated health.

We also examined whether the association between neighborhood types and self-rated health varied with the presence of functional limitations. As Table 4 shows, residing in different neighborhood types was not significantly associated with self-rated health for older adults without functional limitations. However, neighborhood type was significantly associated with self-rated health for older adults with functional limitations. Specifically, those with functional limitations who lived in neighborhoods with limited services (class 1) reported poorer self-rated health compared to those residing in neighborhoods characterized by supportive social engagement (Class 4) (b = −0.36, *p* = 0.001). Similarly, living in service-integrated areas (Class 2) was associated with lower self-rated health compared to living in socially supportive neighborhoods (b = −0.33, *p* < 0.001). There was no association between the healthy environment zone (Class 3) and supportive social engagement (Class 4).

## 4. Discussion

Interest in the congruence between individuals’ intrinsic capacities and their physical and social resources has increased across social science disciplines since the early twentieth century. Lawton and Nahemow proposed the concept of “person–environment fit” to suggest that older adults’ well-being depends on the goodness-of-fit between their abilities, the physical and social aspects of the environments in which they live, and high-quality preventive, supportive, and compensatory services [23].

While planned communities are developed with residents’ needs in mind, older adults who reside in neighborhoods experience a wide, variable, and fluctuating array of physical and psychosocial needs and resources [37]. The current study advances knowledge about congruence in the needs and resources of community-dwelling older adults. Adding specificity and conceptual clarity to the WHO Age-Friendly Cities and Communities (AFC) framework, we used the AARP’s seven “livability indices” to identify a four-class typology of neighborhood types: “Connected yet Limited Services”, “Service Integrated”, “Healthy Environment Zones”, and “Supportive Social Engagement”. We determined the proportion of older adults in each type of neighborhood and examined the association of neighborhood type with older adult residents’ self-rated health.

Neighborhoods feature a diverse mix of characteristics—some excel in transportation but lack in housing, for example, while others offer better social environments but weaker physical environments. No neighborhood perfectly integrates all built, social, and service environments, which highlight the complexity of neighborhood classifications. “Connected yet Limited Service” neighborhoods excel in housing (i.e., affordability and access), environment (i.e., clean air and water), social engagement (i.e., civic and social involvement), and opportunities (i.e., inclusion and possibilities) but lack sufficient resources (i.e., access to life, work, and recreation), transportation (i.e., safe and convenient options), and health services (i.e., access to health care and quality of care). “Service-integrated” neighborhoods have a well-established built environment, including quality housing, resources, and transportation, along with quality health services. Yet, they are weaker in terms of the natural environment and social engagement. “Healthy Environment” neighborhoods provide a high-quality natural environment and health services, but built environments and social engagement are underdeveloped. Finally, “Supportive Social Engagement” neighborhoods afford residents better quality natural environments, health services, and social environments but limited built environments.

As is consistent with previous literature, we found that age, race/ethnicity, socioeconomic indicators, and social interaction were associated with better self-rated health while living with a spouse or partner and having physical limitations were associated with worse self-rated health. Older adults who live in “connected yet limited services” and “service-integrated” neighborhoods reported worse self-rated health than those in neighborhoods characterized by supportive social engagement. Likewise, older adults with physical limitations who resided in neighborhoods with limited services and service-integrated areas reported poorer self-rated health than those in socially supportive neighborhoods. These findings support Lawton and Nahemow’s ecological theory of aging [23], which emphasizes the crucial role of the interaction of an individual’s abilities and environmental resources in their well-being. Our results also highlight the importance of ensuring suitable environments for more vulnerable populations, such as older adults who have functional limitations, and access to appropriate social and physical resources.

Identifying the relative strengths and weaknesses of different neighborhoods is important for theoretical, empirical, and applied reasons. This information can be used to conceptualize and refine knowledge about the dynamics of current and changing neighborhood characteristics vis-à-vis the strengths and needs of older adults. It can also inform research on the mechanisms, including direct and indirect pathways, that promote and preserve physical health and other aspects of well-being. Practically speaking, understanding the fit between needs and resources in a given neighborhood is essential for the effective efficient planning and delivery of services. Finally, since the composition of neighborhoods and the mix of factors that make them more or less age-friendly change over time, the adequacy of fit should be periodically reassessed. Our findings suggest, for example, that older adults with functional limitations may need more health support to enable them to age in place.

This study highlights the importance of quality health services and natural environments (i.e., clean air and water) for older adults’ health, without which the benefits of built and social environments are diminished. More effective guiding strategies are necessary to address older adults’ needs. For example, the WHO’s “Urban Health Initiative” aims to improve health conditions by reducing air pollution in low-income urban neighborhoods [38], where more than 90% of residents live with polluted air and the WHO provides a guiding tool for integrating such health measures into policymaking [39]. Furthermore, successful strategies require active contributions from diverse stakeholders across multiple sectors and professions, including government, nonprofit, and private sectors, especially given the federal government’s limited role in funding AFC initiatives in the United States [40].

Age-friendly communities must support older adults’ right to a meaningful life without regard for personal competencies [40]. Yet older racial and ethnic minority adults and those living in low-income neighborhoods tend to face more disadvantages [41]. Targeting these populations can be beneficial. For example, a university–community partnership, the Richmond Health and Wellness Program (RHWP) in Virginia, targets low-income senior housing communities in the Greater Richmond Area, providing weekly wellness visits based on holistic care coordination; these visits have improved residents’ quality of life [42]. Healthcare professionals conduct health assessments, identify care gaps, and offer wellness coaching. The program’s advisory council advances social equity by addressing race- and culture-related issues [43]. Future evaluations are needed to determine how well the program meets the unique needs of older adults in low-income senior housing.

This study has several limitations. The cross-sectional design limits our ability to infer causal relationships between neighborhood types and health outcomes. In addition, the sample focuses on specific metropolitan areas in the U.S., which may not represent rural areas or smaller towns where livability factors differ. Although indices are commonly used to conceptually summarize data, they are not without limitations. Examples include potential bias in weighting different indicators, the oversimplification of concepts that leads to an inability to adequately capture complexity and context; and data-related matters such as missing data, overlooking within-sample subgroup variation, and concerns about stability over time. We used zip codes as a proxy for the neighborhood. In rural areas, multiple municipalities may share the same zip code, which can complicate findings. However, using zip codes provides practical implications for addressing the narrower scope of neighborhood services as well as considering data availability.

The bivariate analyses indicated that older adults with higher socioeconomic status (SES) are more likely to reside in neighborhoods with supportive social engagement. These findings highlight structural inequalities embedded in residential choices and the distribution of neighborhood resources [44]. Although the main analyses are statistically adjusted for SES, the health effects of neighborhood environments may vary according to an individual’s SES. Future research should examine the interaction between SES and neighborhood types to uncover the potential compounding effects of individual and environmental disadvantages. Finally, the relationship between race/ethnicity and neighborhood types is more complex than that between socioeconomic status and neighborhood types. The bivariate analyses show that older adults live in various neighborhood types rather than a single type. For instance, older White adults tend to reside in neighborhoods with either connected yet limited services or supportive social engagement, while older Hispanics live in service-integrated or healthy environment zones. The study sample is skewed to white older adults (over 80%), which affects the generalizability of health associations to other racial/ethnic groups. In future research, we will investigate the effects of interactions between race/ethnicity and neighborhood types on health. Additionally, subsequent studies should investigate whether the four distinct neighborhood types are consistent across different samples, locations, and time periods.

Community organizations face significant challenges in assessing the impact of AFC initiatives, largely due to limited multi-year funding for evaluations. Progress has been made in strategic management structures, the application of age-friendly frameworks, reported activities, and multi-sector collaboration [45], but evaluative evidence remains weak, most notably in monitoring individual and community outcomes and addressing barriers [46]. Menec et al. found that even successful AFCs, such as those in Manitoba, Canada, struggled with evaluations due to a lack of secure funding and reliable support [47]. These challenges threaten the sustainability of AFC initiatives and hinder their ability to fulfill the WHO’s goal of enhancing older adults’ health and well-being by improving the quality of built and social environments and providing essential services [10,48]. Overcoming these issues will require actionable strategies based on the complex, multifaceted, and dynamic features of neighborhoods.

## 5. Conclusions

This study provides important new insights into the combined effects of livable community domains on older adults’ self-rated health. Notably, older adults with functional limitations who live in neighborhoods with limited services reported poorer self-rated health than those in socially supportive neighborhoods, while service-integrated neighborhoods were associated with lower self-rated health compared to socially supportive ones. These findings highlight the need to foster environments that promote meaningful social engagement alongside accessible services and, more broadly, to develop targeted strategies that align with the multifaceted nature of AFCs and their varying impacts on older adults’ well-being. Collaborative efforts to ensure equitable access to high-quality healthcare and opportunities for social engagement are crucial for enhancing the sustainability of AFC initiatives and promoting the health and well-being of older adults and aging populations.

## Figures and Tables

**Table 1:** Model selection criteria for neighborhood types.

Model	AIC	BIC	Entropy	VLMR-LRT
1 Class	27,700.110	27,742.631		
2 Classes	25,606.081	25,697.196	0.857	*p* < 0.001
3 Classes	24,106.180	24,245.890	0.968	*p* < 0.001
4 Classes	23,405.456	23,593.761	0.936	*p* < 0.001
5 Classes	23,018.036	23,254.935	0.925	*p* < 0.001

Note: AIC = Akaike information criterion, BIC = adjusted Bayesian information criterion, VLMR-LRT = Vuong–Lo–Mendell–Rubin adjusted likelihood ratio test.

**Table 2:** Sample characteristics by neighborhood type.

		Neighborhood Type				
	Total (n = 3211)	Connected Yet Limited Services (n = 509)	Service Integrated (n = 897)	Healthy Environment Zone (n = 762)	Supportive Social Engagement (n = 1043)	Statistic
Age						*x*^2^ = 18.79 **
65–74, %	57.52	57.37	59.75	58.92	54.65	
75–84, %	27.75	29.86	28.21	26.77	27.04	
85 and older, %	14.73	12.77	12.04	14.30	18.31	
Sex (Female), %	59.02	62.67	59.98	56.30	58.39	*x*^2^ = 5.65
Race/ethnicity						*x*^2^ = 216.32 ***
White, non-Hispanic, %	80.69	87.65	74.83	78.37	91.78	
Black, non-Hispanic, %	6.48	9.36	8.55	7.35	3.27	
Hispanic, %	7.63	0.60	15.24	12.52	1.78	
Other race, non-Hispanic, %	2.15	2.39	1.39	1.77	3.17	
Education (college), %	47.24	37.03	45.21	47.38	56.78	*x*^2^ = 58.81 ***
Income, M	4.41	3.97	4.23	4.43	4.77	*F* = 21.77 ***
Employment (yes), %	16.66	15.35	15.18	17.65	18.56	*x*^2^ = 5.06
Own home (yes), %	82.75	88.17	77.79	86.45	82.55	*x*^2^ = 33.42 ***
Living with spouse/partner, %	49.08	47.91	49.55	49.80	50.78	*x*^2^ = 1.13
Five years in community (yes), %	93.09	93.91	93.98	90.55	93.77	*x*^2^ = 10.01 *
Social interaction, M	5.06	5.10	5.05	5.04	5.08	F = 0.39
Physical limitation (yes), %	28.99	35.50	29.12	29.55	26.63	*x*^2^ = 12.94 **
Caregiver (yes)	15.01	15.38	15.46	16.13	14.19	*x*^2^ = 1.38

Note: * *p* < 0.05, ** *p* < 0.01, *** *p* < 0.001.

**Table 3:** Relationship between neighborhood type and self-rated health.

	Estimate	S.E.
Age 85 and older (ref)		
65–74	−0.19 ***	0.05
75–84	−0.22 ***	0.06
Sex (Female)	0.04	0.04
Race/ethnicity		
White (ref)	-	
Black	−0.22 **	0.07
Hispanic	−0.15 *	0.07
Other race	−0.14	0.13
Education (college)	0.14 ***	0.04
Income	0.07 ***	0.01
Employment (yes)	0.20 ***	0.05
Own home (yes)	0.12 *	0.05
Living with spouse/partner	−0.12 **	0.04
Five years in community (yes)	−0.09	0.07
Social interaction	0.06 ***	0.01
Physical limitation (yes)	−0.95 ***	0.04
Caregiver (yes)	0.02	0.05
Neighborhood type		
Connected yet limited services	−0.21 ***	0.06
Service integrated	−0.16 ***	0.05
Healthy environment zone	−0.05	0.05
Supportive social engagement (ref)	-	-

Note: * *p* < 0.05, ** *p* < 0.01, *** *p* < 0.001.

**Table 4:** Relationship between neighborhood type and self-rated health by functional limitation.

	No Functional Limitations (n = 2232)		Functional Limitations (n = 931)	
	Estimate	S.E.	Estimate	S.E.
Connected yet limited services	−0.13	0.07	−0.36 **	0.10
Service integrated	−0.10	0.05	−0.33 ***	0.09
Healthy environment zone	−0.01	0.05	−0.09	0.10
Supportive social engagement (ref)			-	-

Note: ** *p* < 0.01, *** *p* < 0.001.

## Data Availability

The data presented in this study are available on request from the corresponding author due to privacy.
